# A comprehensive framework for the management of hereditary breast cancers: guiding light in precision medicine

**DOI:** 10.3389/or.2025.1633387

**Published:** 2025-09-19

**Authors:** Samar Ekram, Mariam M. Al Eissa

**Affiliations:** ^1^ Department of Medical Genetics, Faculty of Medicine, Umm Al-Qura University, Makkah, Saudi Arabia; ^2^ Department of Medicine, Medical School, Alfaisal University, Riyadh, Saudi Arabia; ^3^ Molecular Genetics Laboratory, Public Health Authority, Public Health Lab, Molecular Genetics Department, Riyadh, Saudi Arabia; ^4^ The Computational Sciences Department at the Centre for Genomic Medicine (CGM), King Faisal Specialist Hospital & Research Center, Riyadh, Saudi Arabia; ^5^ King Khaled Eye Specialist Hospital (KKESH) Research Department, Riyadh, Saudi Arabia

**Keywords:** precision medicine, hereditary breast cancer, HBC, genetic testing, Saudi Arabia, cancer prevention

## Abstract

**Background:**

The landscape of oncology varies across countries and regions, and in consanguineous populations such as Saudi Arabia, the clinical management of hereditary cancers poses a distinct challenge. Hereditary breast cancer (HBC), which is a significant public health concern, accounts for approximately 5%–10% of all breast cancer cases. High-risk genes, including BRCA1, BRCA2, PALB2, TP53 and PTEN, with germline pathogenic or likely pathogenic variants (PVs/LPVs), substantially increase the risk of breast cancer and other malignancies.

**Method:**

In this review, we explore the guidelines and the literature to present a comprehensive investigation of the genetic landscape of hereditary cancer syndromes, provide pivotal insights into disease mechanisms and inform precise clinical intervention. Given their marked therapeutic heterogeneity, a tailored precision medicine approach, rather than a uniform strategy of a one-size-fits-all model, is necessary. For high-risk breast cancer patients in Saudi Arabia, the detection rates of PVs/LPVs have reached 24%, underscoring the relevance of targeted interventions.

**Results:**

A comprehensive framework for the management of HBCs is outlined, which focuses on consanguineous populations and adapts global guidelines. We highlight the critical roles of genetic testing in guiding personalised surveillance strategies, especially for regions where data remain limited.

**Conclusion:**

Revealing the genetic variation associated with HBCs mitigates the burden on healthcare providers and the long-term effects of HBCs on affected individuals and their families. Moreover, it is a step ahead towards personalised prevention, treatment and intervention. This knowledge will empower research and innovation in biotechnology.

## Introduction

### Hereditary breast cancer

One of the major public health concerns is hereditary breast cancer (HBC), a genetic condition in which genetic factors significantly contribute to the development of breast cancer (BC), accounting for 5%–10% of all BC cases, and approximately 25%–40% of BC cases occur i 35-year-old females (1). Breast cancer is characterised by a higher-than-normal risk of developing into breast cancer. Germline pathogenic or likely pathogenic variants PVs/LPVs of any breast cancer predisposing genes (*BRCA1, BRCA2, PALB2, TP53, PTEN, CDH1, CHEK2, ATM* and others) can lead to this condition (2, 3). Mutations in the BRCA1 and BRCA2 genes lead to chromosomal instability and increase the risk of having cancer, accounting for 3%–8% of BC cases and 30%–40% of HBC cases (1, 4).

In Saudi Arabia, the incidence of BC is gradually increasing, with an earlier age of onset and with the age-standardised incidence rate increasing from 11.8 to 29.7 per 100,000 population, thus making it a more advanced disease compared with global trends. About 55%–75% of cases are diagnosed before the age of 50, in contrast to approximately 30% in Western countries (5). This epidemiologic pattern suggests that underlying genetic predispositions may contribute significantly to disease aggressiveness. The high rate of consanguinity within the population has led to increased genetic homogeneity, which may facilitate the accumulation of deleterious germline variants, including potential founder mutations, factors that likely contribute to the growing burden of HBC in the region (6, 7). Compounding this issue is the limited availability of genetic screening programmes and the under-recognition of hereditary cancer syndromes, which continue to obscure the true prevalence at the national level. Although population-based data are lacking, studies conducted in high-risk cohorts, such as those with early-onset disease or a strong family history, have reported PV/LPV detection rates as high as 24%, reflecting a substantially elevated hereditary burden within these selected groups (7). These findings point to an urgent need for broader access to genetic services, more effective early detection programmes and well-designed population-based research to clarify the full extent of hereditary cancer risk in the community.

Genetic testing for HBC syndrome is recommended for individuals who meet specific criteria, including the following: known PV/LPV mutation in one of the BC predisposing genes in the family; BC detected at or before the age of 50; triple-negative BC; lobular BC with a personal or family history of diffuse gastric cancer (DGC); treatment indications (to inform the use of poly (ADP-ribose) polymerase [PARP] inhibitors in the treatment of breast, ovarian, prostate and pancreatic cancer), personal and/or family history indicating a potential genetic predisposition to breast cancer; male BC; ovarian cancer; ethnic or geographic ancestry associated with a higher prevalence of BRCA mutations, such as Ashkenazi Jewish descent; and individuals with a probability >5% of a *BRCA1/2* P V/LPV based on prior probability models (e.g., Tyrer-Cuzick, BRCA Pro and CanRisk) (8).

Genetic counseling plays a crucial role in mitigating, managing, and understanding hereditary cancer risk, as well as facilitating evidence-based decision-making. In individuals with HBC, strategies for surveillance are tailored based on the top gene mutation identified and the pathogenic variant identified, with risk stratification into high (>40%), moderate (20–40) and low risk (≲20) categories (Table 1) (9–11), guiding the scope of clinical monitoring. Tailoring surveillance to a specific risk level helps optimise early detection and intervention (Table 2–4). International recommendation guidelines regarding surveillance vary between countries and regulatory bodies, such as those from the European Society for Medical Oncology (ESMO), National Comprehensive Cancer Network (NCCN) and others, which should consider the context of regional practices, population variations and available resources (8–10, 12–14).

**TABLE 1:** Gene susceptibility with risk estimates for HBCs across high-, moderate- and low-risk carriers.

Risk category	Genes	Approximate lifetime breast cancer risk
High risk	*BRCA1, BRCA2, PALB2, TP53, PTEN, CDH1*	>40%
Moderate risk	*ATM, CHEK2*	20%–40%
Low risk	*RAD51C, RAD51D, others*	≲20%

**TABLE 2:** Cancer risk management strategies for high-risk genes.

High-risk genes (BRCA1, BRCA2, PALB2, TP53 and PTEN)					
Gene	Surveillance	Starting age	Modalities	Additional measures	Preventive surgery
BRCA1 and BRCA2	Breast cancer surveillance (8, 14)	Begin at age 25 or earlier if there is a family history of early-onset breast cancer	Annual MRI with and without contrast starting at age 25 and supplemented with mammography at age 30. Consider alternating MRI and mammography every 6 months for thorough surveillance	Breast cancer awareness at age 18 years Clinical breast exams every 6–12 months starting at age 25	Risk-reducing mastectomy (RRM) significantly lowers the risk of breast cancer by approximately 90%–95%
	Surveillance for other cancers (25, 51, 52)				
	Ovarian cancer surveillance (8, 53)	Begin at age 35–40 or earlier if there is a family history of early-onset ovarian cancer	No effective screening method for ovarian cancer	Transvaginal ultrasound and CA-125 blood tests (They are not highly effective in the early detection of the disease but can be considered alternatives to prophylactic salpingo-oophorectomy for patients who either refuse surgery or are not suitable candidates.) Frequency: Every 6–12 months	Prophylactic salpingo-oophorectomy is recommended after childbearing is complete or by age 35–40 for BRCA1 mutation carriers and 40–45 for BRCA2 mutation carriers, dramatically reducing ovarian and breast cancer risk in perimenopausal women. Salpingectomy with delayed oophorectomy can be considered in younger patients
	Prostate cancer surveillance (8, 12)	Begin at age 40	Annual PSA level and baseline digital rectal examination are recommended for BRCA2 mutation carriers and can be considered for BRCA1 mutation carriers		
	**Pancreatic cancer surveillance** (48)	Individuals with mutations and a family history of pancreatic cancer should consider starting at age 50 or earlier if there is a family history of early-onset pancreatic cancer	Annual surveillance with endoscopic ultrasound (EUS) and/or contrast-enhanced magnetic resonance cholangiopancreatography (MRCP)		
PALB2	Breast cancer surveillance (8, 14)	Begin at age 25 or earlier if there is a family history of early-onset breast cancer	Annual breast screening using MRI with and without contrast starting at age 25, supplemented with mammography beginning at age 30. Consider alternating MRI and mammography every 6 months for thorough surveillance	Breast cancer awareness at age 18 Clinical breast exams every 6–12 months starting at age 25	RRM significantly lowers the risk of breast cancer by 90%–95%
	Surveillance for other cancers (24, 51)				
	Pancreatic cancer surveillance (48)	Individuals with mutations and a family history of pancreatic cancer should consider starting at age 50 or earlier if there is a family history of early-onset pancreatic cancer	Annual surveillance with EUS and/or contrast-enhanced MRCP		
TP53	Breast cancer surveillance (12, 51)	Begin at age 20–25 or earlier if there is a family history of early-onset breast cancer	Annual breast MRI with and without contrast at the age of 20. Annual mammograms are recommended starting at age 30 and continuing as a complementary modality to MRI for enhanced detection	Breast cancer awareness at age 18 Clinical breast exam every 6–12 months starting at age 20	It is an option for high-risk breast cancer to perform RRM, which significantly lowers the risk of breast cancer
	Surveillance for other cancers (12, 51)				
	Surveillance for a wide range of malignancies, including soft tissue and osteosarcomas, brain tumours, adrenocortical carcinoma, colorectal and other solid and haematologic malignancies	Begin in infancy	1) An annual whole-body MRI to detect early-stage solid tumours across various body regions (51) 2) Annual brain MRI scans to monitor central nervous system neoplasms 3) Semi-annual screening of abdominal and pelvic ultrasound should be performed to detect adrenocortical carcinomas and other abdominal malignancies 4) Regular blood tests, including complete blood counts, to detect hematologic malignancies, such as leukaemia and lymphoma 5) Colonoscopy and upper endoscopy every 2–5 years starting at age 25 or 5 years before the earliest known colorectal or gastric cancer in the family, respectively	A comprehensive physical examination, including dermatological and neurologic assessment, should be conducted every 6–12 months with a high level of vigilance for rare cancers and second malignancies in cancer survivors	
PTEN	Breast cancer surveillance (8, 14, 54)	Begin at age 30 or earlier if there is a family history of early-onset breast cancer	Annual breast mammogram and MRI with and without contrast starting at age 30. Consider alternating MRI and mammography every 6 months for thorough surveillance	Breast cancer awareness at age 18 Clinical breast exam every 6–12 months starting at age 25	RRM significantly lowers the risk of breast cancer by 90%–95%
	Surveillance for other cancers (54)				
	Thyroid, colon, endometrial, renal and skin cancers	Begin during childhood	1) Annual thyroid ultrasounds starting at age 7 2) Colonoscopy every 5 years starting at age 35 or earlier if symptomatic or if a close relative had colorectal cancer before age 40, with adjustments based on findings and the recommendations of a gastroenterologist 3) Recommended renal ultrasound screening every 2 years, starting at age 40	A comprehensive physical examination is recommended to be performed annually starting at age 18 or if a family history is reported 5 years before the youngest age of cancer diagnosis. Particular attention should be given to thyroid, dermatological and neurological evaluations during these exams/ Routine surveillance for endometrial cancer is not typically recommended but may be considered on an individual basis. Patients should be advised to report abnormal uterine or postmenopausal bleeding promptly, as these could indicate early-stage endometrial cancer **Paediatric considerations:** Neurodevelopmental, dermatological and thyroid evaluations should be performed early and regularly	A prophylactic hysterectomy after childbearing is complete can be considered
C**DH1**	Breast cancer surveillance (51)	Begin at age 30 or earlier if there is a family history of early-onset breast cancer	Annual breast mammogram and MRI with and without contrast starting at age 30. Consider alternating MRI and mammography every 6 months for thorough surveillance	Breast cancer awareness at age 18 Clinical breast exam every 6–12 months starting at age 25	RRM may be considered
	Surveillance for other cancers				
	G**astric** cancer surveillance (51)	Begin at age 20–25 or earlier if there is a family history of early-onset gastric cancer	Annual endoscopic screening with targeted and random biopsies		Risk-reducing gastrectomy maximises risk reduction for advanced gastric cancer and gastric cancer mortality to <1%

### High-risk genes (BRCA1, BRCA2, PALB2, TP53, PTEN and CDH1)

#### *BRCA1* and *BRCA2* genes

*BRCA1* and *BRCA2* are tumour suppressor genes that play critical roles in maintaining genomic integrity by facilitating DNA repair through homologous recombination. Pathogenic alterations in these genes lead to impaired mechanisms of DNA repair, which promote tumorigenesis, particularly in breast and ovarian tissues. Individuals carrying PVs or LPVs in these genes exhibit significantly elevated risks over their lifetimes for breast and ovarian cancer. Specifically, *BRCA1* mutations confer a lifetime BC risk of 72% and are 44% associated with ovarian cancer risk. In comparison, *BRCA2* mutations confer a lifetime risk of up to 69% for BC and 17% for ovarian (15–17). On a global scale, approximately one in 400 individuals is estimated to carry a mutation in either the *BRCA1* or *BRCA2* gene, although the prevalence of *BRCA* mutations varies considerably across different populations. The significantly greater prevalence of certain populations is due to genetic factors, such as founder effects. For example, the likelihood of Ashkenazi Jewish individuals carrying a *BRCA1* or *BRCA2* mutation is one in 40; specific founder mutations increase this prevalence and have persisted and proliferated over generations within the Ashkenazi Jewish population (18).

In Saudi Arabia, BC is the most frequently diagnosed malignancy among women. Recent research has indicated that mutations in the *BRCA* gene contribute significantly to the burden in the region for HBC and ovarian cancer. One study revealed that 8.7% of Saudi BC patients carry *BRCA* mutations, with *BRCA1* mutations being more prevalent than *BRCA2* mutations (19, 20). This high prevalence underscores the importance of incorporating genetic risk into the clinical management of patients with BC and ovarian cancer in Saudi Arabia. Given the substantial risk conferred by *BRCA* mutations, counseling and genetic testing are essential components of cancer prevention, early intervention and personalised medicine strategies. The early identification of individuals with BRCA PVs or LPVs allows for the implementation of targeted preventive measures (Table 2). Research has indicated that risk reduction can reach 90% when prophylactic surgeries, such as salpingo-oophorectomy and bilateral mastectomy, are performed at appropriate ages. For individuals who defer surgery, structured surveillance using annual Magnetic Resonance Imaging (MRI) in combination with mammography offers an alternative approach, with sensitivity rates exceeding 94%, facilitating earlier diagnosis and improved prognosis (21). Aside from prevention, significant progress has been made in the therapeutic management of BRCA-associated malignancies. PARP inhibitors impair DNA repair in tumour cells deficient in homologous recombination. Randomised trials have shown improved clinical outcomes with these agents. For example, treatment with olaparib has extended the median progression-free survival to 11.2 months compared with 4.3 months with standard chemotherapy in patients with germline BRCA mutations (22, 23).

In Saudi Arabia, the prevalence of BRCA mutations among BC patients is noteworthy. To improve the individual risk outcome, genetic screening for BRCA mutations can optimise early detection, early prevention and prompt intervention and provide a targeted treatment by harnessing these genetic insights. Integrating genetic testing and counseling into routine clinical practice is imperative for improving patient outcomes within the region.

#### PALB 2 gene

Significant contributors to cancer susceptibility are increasingly recognised in *PALB2* mutation carriers, the mutations of which are found in breast and pancreatic cancers. The gene works as a partner and linker of *BRCA2*, which plays a crucial role in DNA repair through homologous recombination, a process essential for maintaining genomic stability. Several studies have identified a significantly elevated risk of developing BC in carriers with heterozygous germline mutations in *PALB2*, with a lifetime risk estimate of 33%–58% by age 70 (16, 17). This risk is comparable with that associated with *BRCA2* mutations, underscoring the importance of *PALB2* in hereditary BC syndromes. In addition, mutations in the *PALB2* gene confer an increased risk of different types of cancers, particularly pancreatic cancer. Although data on pancreatic cancer risk are more limited, current evidence indicates a lifetime risk of approximately 2%–3% in *PALB2* carriers, which is significantly higher than that of the general population (8). There is also accumulating evidence supporting a potential link between *PALB2* mutations and other cancers, such as ovarian and prostate cancer, highlighting the broader clinical significance of *PALB2* in hereditary cancer syndromes. In Saudi Arabia, studies on the *PALB2* mutation in BC patients are limited, but this mutation seems to be present at a very low frequency of 0.65% (20, 25).

The cancer risk associated with *PALB2* mutations within a gene and the type and location of the mutation strongly influence the associated risk. Truncating mutations are particularly deleterious because they create shortened and non-functional proteins. Missense mutations can also be pathogenic or non-pathogenic, depending on their interaction with *BRCA one* and B*RCA two* or their location, which influences the protein’s structure. To determine the clinical significance of specific *PALB2* variants, functional assays and genetic counselling are essential. The surveillance recommendations for individuals with PALB2 gene mutations focus primarily on early detection and prevention of breast and pancreatic cancers and are similar to those for BRCA1/2 mutation carriers, given the significantly increased risk associated with these mutations (Table 1 and 2) (8, 26). Furthermore, recognising the role of *PALB2* in the DNA repair pathway is increasingly important, particularly as updated treatment guidelines now recommend assessing this gene when deciding whether patients with breast or pancreatic cancer may benefit from PARP inhibitor therapy (8).

#### *TP53* gene (Li‒Fraumeni syndrome)

Li‒Fraumeni syndrome (LFS) is a hereditary cancer predisposition disorder linked to germline mutations in the tumour suppressor protein p53 (*TP53*) gene. This syndrome is characterised by an increased lifetime risk of developing multiple primary cancers, often at an early age, including childhood malignancies (27). Mutations in this gene are found in 2%–6% of BC patients under the age of 35 (28). However, LFS is associated with a spectrum of malignancies, including but not limited to breast cancer, soft tissue sarcomas, osteosarcomas, brain tumours, adrenocortical carcinomas (ACC) and hematologic malignancies such as leukaemia. Females with a TP53 mutation had a 50% chance of developing cancer by the age of 31, while males reached a similar risk by the age of 46, according to the National Institutes of Health. By age 70, individuals of both genders have nearly 100% cumulative cancer risk (29). In *TP53* mutation carriers, the cumulative incidence by age 70 is estimated at 54% for breast cancer, 15% for soft tissue sarcoma, 6% for brain tumours and 5% for osteosarcoma (30).

Therefore, identifying these individuals is crucial by following the NCCN testing criteria for Li–Fraumeni syndrome: an individual from a family known to carry (P/LP) the *TP53* variant; individuals diagnosed before age 46 with cancer from the LFS spectrum (e.g., soft tissue sarcoma, osteosarcoma, central nervous system tumour, breast cancer and ACC); individuals having at least one first- or second-degree relative diagnosed with any of these cancers (excluding BC if the proband has breast cancer) before age 56 or with multiple primary cancers at any age; individuals with multiple tumours (excluding multiple breast tumours), with at least two from the LFS spectrum where the initial cancer occurred before age 46; individuals diagnosed with ACC, choroid plexus carcinoma or embryonal anaplastic subtype rhabdomyosarcoma at any age, regardless of family history; BC diagnosed before age 31; and personal or family history of paediatric hypodiploid acute lymphoblastic leukaemia (8, 31, 32).

Despite the well-established criteria for diagnosing LFS, many patients with TP53 mutations do not meet these criteria. This may be due to the high rate of *de novo* TP53 mutations, which have an incidence of 20% (33). In Saudi Arabia, germline *TP53* pathogenic mutations are detected in 1.5% of early-onset BC patients. Most of these patients do not have a family history indicative of LFS or a personal history of multiple LFS-related tumours (34). However, studies in Saudi Arabia that accurately reflect the incidence and cancer risk associated with this syndrome are lacking.

Approximately 70% of individuals diagnosed with LFS exhibit a pathogenic *TP53* germline variant. However, the remaining 30% of patients do not carry a *TP53* variant, and even among those with the variant, approximately 20% remain cancer-free (35). Understanding variability in cancer penetrance and the diverse clinical manifestations of LFS is crucial for developing precise approaches for early tumour detection and effective strategies for reducing cancer risk. For example, BC patients with a PV/LPV in the *TP53* gene are usually treated with mastectomy instead of lumpectomy to avoid potential radiation-induced malignancies (36).

Moreover, it is important to recognise that somatic *TP53* variants, which occur in non-germline cells, frequently confound germline testing results. Coffee et al. reported that 38.8% of *TP53* PVs identified through a commercially available hereditary cancer panel were likely somatic, rather than germline, variants (37). Therefore, it is crucial to recognise when a PV is somatic rather than germline and to distinguish between somatic mosaicism and clonal haematopoiesis (38). Somatic mutations in *TP53* are commonly observed in a wide spectrum of malignancies. Consequently, this complicates diagnosis when genetic testing is performed in older individuals and/or cancer patients. Therefore, a careful interpretation of genetic test results is essential, considering the patient’s clinical context and family history (39).

Considering the markedly increased cancer risk associated with LFS, the adoption of a rigorous and comprehensive surveillance system protocol is essential to facilitate the early detection of tumours and to enhance patients’ clinical outcomes. Surveillance typically begins in early childhood, often by the first year of life, according to the American Association for Cancer Research and NCCN guidelines (Table 2) (25, 40).

## PTEN gene (cowden syndrome)

Cowden syndrome (CS) is a rare genetic disorder known to affect approximately one in 200,000 individuals worldwide. First identified in 1963, this autosomal dominant disorder is associated with mutations in the phosphatase and tensin homolog (*PTEN*) gene, which is located at chromosome 10q23.3. The *PTEN* gene functions as a tumour suppressor primarily by inhibiting the PI3K/Akt/mTOR signalling pathway, which regulates critical cellular processes, such as growth, proliferation, angiogenesis and survival (41, 42).

Individuals with CS typically present with macrocephaly and are at a heightened risk for both benign and malignant tumours across various organs, particularly the breast, thyroid and endometrium. Benign lesions that are common include those affecting the skin, colon and thyroid. Mucocutaneous manifestations, intestinal polyps, hamartomatous overgrowth and vascular anomalies are also characteristic of the syndrome (43). However, the diagnosis can be complicated due to variable expressivity and incomplete penetrance (approximately 80%) (43).

The timely identification of CS is essential to enable appropriate cancer screening and intervention to reduce the risk of associated complications. However, despite substantial research efforts, genotype–phenotype correlations in CS remain inadequately elusive. Some studies have suggested that patients with missense *PTEN* mutations may have a lower risk of thyroid cancer, mutations in the promoter region of the gene may increase BC risk, and nonsense mutations may increase the risk of colorectal cancer (44, 45).

Women with CS face a significant risk of both benign and malignant breast diseases, often concurrent with other syndrome features. The lifetime risk of BC for *PTEN* mutation carriers is approximately 85%, and these patients frequently present with bilateral disease. This risk is comparable with that seen in hereditary breast and ovarian cancer syndrome, thus justifying the need for similar high-risk screening and surgical interventions (8, 46).

Thyroid disease is another significant concern for *PTEN* mutation carriers, with an estimated 88% of patients developing benign lesions and 35% experiencing malignant thyroid conditions. Childhood-onset thyroid cancers have been reported, with the youngest being diagnosed at 7 years of age and other cases occurring at ages 11 and 13 (8).

Hamartomatous and mixed gastrointestinal polyps are commonly observed in CS patients, increasing the risk of colorectal cancer. Although colorectal cancer is rare in this population, gastrointestinal surveillance is critical to reduce polyp burden and prevent malignancy (8).

Women with *PTEN* mutations are also at risk for benign endometrial lesions (e.g., fibroids) and benign renal lesions. The lifetime risk of endometrial cancer is estimated at 28%, typically emerging in the late 30s or early 40s, while the risk of renal cell carcinoma is approximately 35% in patients over 40 years of age (8).

Vascular anomalies have also been linked to *PTEN* mutations, which are attributed to the loss of the angiogenic regulatory function of *PTEN*. The true frequency and spectrum of these anomalies are likely underestimated given their varied presentations in different organs (47). The most common cerebral vascular anomalies in *PTEN* PV or LPV carriers are benign developmental venous anomalies, with dural arteriovenous fistulas also frequently documented. However, no clear genotype‒phenotype correlation has been established between the type of vascular anomaly and the specific mutation (47).

The most serious consequence of *PTEN* mutations is increased cancer risk, particularly in the breast, thyroid, endometrium and kidneys, with a lower but notable risk for colorectal cancer and melanoma. Once a diagnosis of CS is confirmed, the primary focus of management is vigilant surveillance to detect tumours at an early stage, which is the treatable stage (Table 2). Prophylactic surgeries may also be considered in some cases to reduce cancer risk (47).

### CDH1 gene

The *CDH1* gene encodes E-cadherin, a protein critical for maintaining the integrity and functionality of epithelial tissues. This protein plays a key role in promoting cell‒cell adhesion, thereby preventing the cellular detachment and migration processes fundamental to cancer metastasis (48). Pathogenic variants in the *CDH1* gene have been identified as significant contributors to hereditary diffuse gastric cancer and invasive lobular breast cancer. The identification and management of individuals with *CDH1* gene mutations is critical for reducing cancer risk, particularly in regions such as Saudi Arabia, where genetic cancer susceptibility to breast cancer warrants further attention (40).

Globally, germline pathogenic mutations in the *CDH1* gene are relatively rare, with an estimated prevalence of one in 100,000 individuals in the population. Nevertheless, these mutations confer a significantly elevated cancer risk. The estimated lifetime penetrance of *CDH1* mutations is substantial: individuals may develop DGC in up to 42% of men and 33% of women. Female carriers also have a 37%–55% lifetime risk of developing invasive lobular breast cancer (8).

In the Saudi context, emerging data indicate a growing recognition of genetic contributions to predisposition in hereditary cancer syndromes, including those associated with *CDH1* mutations. Prevalence and impact are difficult to comprehensively evaluate because of the scarce data available. This highlights the gap in data documentation through databases and research in the region to provide a better understanding of the role of *CDH1* mutations in cancer predisposition. AlHarbi et al. (49) examined the role of *CDH1* and other cancer-predisposing genes and emphasised the need for tailored genetic screening and interventions in the Saudi population. This was also emphasised by Abdel-Razeq et al., who proposed that a comprehensive genetic screening programme for the Gulf region could provide a better understanding of mutation prevalence and its clinical implications (50).

### Moderate-risk genes and breast cancer susceptibility: a focus on *ATM* and *CHEK2*

Susceptibility to BC is influenced by various genetic factors, ranging from high-risk mutations to more moderate-risk variants. Genes such as *BRCA1* and *BRCA2,* which have high penetrance, have received much attention, while moderate-risk genes such as *ATM* and *CHEK2* are increasingly being known for their considerable percentage in increasing BC risk (Table 1). In a more nuanced and complex manner, these genes contribute to hereditary BC and pose distinct challenges in risk assessment, considerations for genetic counselling and clinical management. Understanding the implications of mutations in *ATM* and *CHEK2* is crucial for guiding the development of personalised surveillance, intervention and risk reduction strategies. However, data and evidence remain sparse, particularly in Saudi Arabia and the wider Middle East, where only a few studies have investigated their prevalence or impact.

### ATM gene

The *ATM* gene encodes a serine/threonine kinase, which plays a critical role in the DNA double-strand break repair pathway. It assesses how genomic stability can be maintained through cell cycle checkpoints and apoptosis activation. Mutations in *ATM* are mainly associated with ataxia-telangiectasia, an autosomal recessive condition characterised by neurodegeneration, immune deficiency and a predisposition to cancer, particularly haematological cancer.

Compared with the general population, heterozygous carriers of *ATM* mutations face an increased BC risk, with studies suggesting a 2–5-fold elevation, translating into a lifetime BC risk of 17%–30% (55). This increased risk stems from the impaired DNA damage repair ability of *ATM* mutation carriers, leading to genomic instability. Although the evidence for *ATM* mutations conferring risks for cancers beyond BC is limited, some studies have suggested potential associations with pancreatic and prostate cancer (52). Bu et al. reported an estimated prevalence of 0.8% for *ATM* germline PVs or LPVs in Arab BC patients who did not carry *BRCA1* or *BRCA2* PVs or LPVs (56). Thus, enhanced BC surveillance is recommended for patients with a confirmed genetic predisposition to breast cancer to facilitate early detection and improve outcomes (Table 3).

**TABLE 3:** Cancer risk management strategies for moderate-risk genes.

Moderate-risk genes (ATM and CHEK2)					
Gene	Surveillance	Starting age	Modalities	Additional measures	Preventive surgery
ATM	Breast cancer surveillance (12, 51)	Begin at age 30–40. Consider earlier screening if there is a family history of early-onset breast cancer or other risk factors	Annual breast mammogram and MRI with and without contrast starting at age 30–40. Consider alternating MRI and mammography every 6 months for thorough surveillance	Clinical breast exam every 6–12 months starting at age 25	For those with a significant family history of breast cancer or additional risk factors, prophylactic mastectomy may be considered
	Surveillance for other cancers (12, 51)				
	Prostate, colon and pancreatic cancer surveillance	Begin at age 4**0** or earlier if there is a family history of early-onset cancer	1) Annual PSA level monitoring and digital rectal exams starting at age 40 can be considered 2) Individuals with mutations and a family history of colon cancer should consider starting colonoscopy every 5–10 years at age 40 or earlier if there is a family history of early-onset colon cancer 3) Routine pancreatic cancer screening is not generally recommended unless there is a significant family history or other risk factors. For these individuals, biennial EUS should start at age 50 or earlier if there is a family history of early-onset pancreatic cancer	**Considerations for homozygous ATM mutations** Regular physical examinations should begin in early childhood, focusing on dermatological assessment and signs of haematologic malignancies to allow for early detection and intervention	
CHEK2	Breast cancer surveillance (12, 51)	Begin at age 30–40. Consider earlier screening if there is a family history of early-onset breast cancer or other risk factors	Annual breast mammogram starting at age 30–40 years, with consideration for adding MRI with and without contrast for enhanced surveillance based on family history and other risk factors. Alternate MRI and mammography every 6 months for thorough surveillance	Clinical breast exam every 6–12 months starting at age 25–30	For those with a significant family history of breast cancer or additional risk factors, prophylactic mastectomy may be considered
	Surveillance for other cancers (12, 51)				
	Colon and prostate cancer surveillance	Begin at age 40 or earlier if there is a family history of early-onset cancer	1) Colonoscopy at age 40 every 5–10 years or earlier if there is a family history of early-onset colon cancer 2) Annual PSA starting at age 40 or earlier if there is a family history of early-onset prostate cancer		

## CHEK2 gene mutation carrier

*CHEK2* encodes a kinase involved in the DNA damage response, primarily by activating *p53*, a pivotal tumour suppressor. Germline mutations in *CHEK2*, particularly the 1100delC variant, have been well established to increase BC risk, with lifetime risk estimates of 23%–27% (57). A significant correlation was found for carriers of truncating *CHEK2* variants with a family history of BC, with a higher prevalence of bilateral BC than of unilateral cases. A 2–4-fold increased risk of BC was estimated for these carriers. However, the absolute risk and likelihood of developing BC at specific ages vary according to additional factors, such as the presence of other genetic susceptibility variants, lifestyle influences and family history, which altogether shape the overall cancer risk for these individuals (58).

In addition to breast cancer, *CHEK2* mutations are linked to an increased risk of other malignancies, including colorectal, prostate and thyroid cancers (58). However, BC remains the most significant clinical concern for *CHEK2* mutation carriers, making it the primary focus of management in clinical practice (52) (Table 3).

## Low-risk genes (*RAD51C* and *RAD51D*)

Data regarding cancer risk management for mutations in moderate- and low-risk genes, such as *RAD51C* and *RAD51D*, are limited compared with those in high-risk genes, such as *BRC one* and *BRCA2*. However, these genes still pose an increased risk of certain cancers, with the absolute risk estimated at 20% and 10%–20% for BC and ovarian cancer, respectively (Table 1) (8). With a lower representation compared with *BRCA1/2* mutation carriers, recent global practice supports the need for tailored screening, prevention and management strategies. An overview of variant representation in the Saudi population correlated with severity and personalised therapy, which assesses refining the clinical guidelines for these mutations (Table 4).

**TABLE 4:** Cancer risk management strategies for low-risk genes.

Low-risk genes (RAD51C and RAD51D)					
Gene	Surveillance	Starting age	Modalities	Additional measures	Preventive surgery
RAD51C and RAD51D	Breast cancer surveillance (51)	Begin at age 40 or earlier if there is a family history of early-onset breast cancer	Annual mammogram at age 40, with MRI considered based on personal and family history	Clinical breast screening every year starting at age 35	For those with a significant family history of breast cancer or additional risk factors, prophylactic mastectomy may be considered
	Surveillance for other cancers (51)				
	Ovarian cancer surveillance	Begin at age 45–50 or earlier if there is a family history of early-onset ovarian cancer	There is no effective screening for ovarian cancer		Prophylactic salpingo-oophorectomy can be considered at the age of 45–50, considering family history and other risk factors

## Health system implications in the Saudi context

In Saudi Arabia, the combination of high rates of consanguinity, the prevalence of early-onset BC and the likelihood of founder mutations highlights the necessity for a well-coordinated, multidisciplinary approach to hereditary cancer care. Currently, services are centralised in tertiary care hospitals, resulting in limited access across various regions. Addressing these disparities requires including genetic results in the current cancer registry in the Saudi Health Council Cancer Registry. This requires wider access to genetic testing and counselling, guaranteed insurance coverage and cost-effective integration to the Saudi Cancer Screening Programme to strengthen prevention, which requires standardised national guidelines and a trained workforce for early detection and personalised care in accordance with Saudi Vision 2030.

## Futuristic innovative approach

Emerging evidence suggests that integrating AI-driven risk stratification models, behavioural science-informed counseling strategies, and culturally contextualised patient engagement can significantly enhance genetic testing programmes in HBC culturally resonant narratives (59, 60). Incorporating principles of behavioural science assess genetic counseling, where it plays a fundamental role as a decision aid, personalised risk framing, where it is shown to increase patient adherence to recommended screening and preventive surgery (61). AI-based in clinical settings can leverage decision support, enabling predictive analytics for resource allocation and flagging high-risk patients for follow-up, increasing cascade testing participation rate (62). The integration of multidisciplinary approaches ensures that precision medicine addresses not only genomic profiles but also both informational and psychosocial determinants of participation. Further assist counsellors by providing dynamic, individualised risk visualisations during sessions.

## Conclusion

Advances in genetic testing have significantly improved the identification and management of HBC. Detecting germline pathogenic variants enables accurate risk stratification, personalised surveillance and targeted preventive measures, ultimately improving outcomes. In Saudi Arabia, where breast cancer incidence is increasing and early-onset disease is common, significant gaps remain in the lack of population-based data representation, with most studies limited to small and high-risk cohorts. This reflects accurate estimates of the potential founder mutations of hereditary cancer prevalence and creates risk models tailored to the Saudi population. This can be addressed by conducting multi-centre research involving broad population sampling and standardised data collection. Future initiatives should include genomic screening empowered by AI analytics and behavioural science, which increases patient engagement strategies, ensuring that precision oncology frameworks are both technologically advanced and socially responsive, tailored to the context of Saudi Arabia culture.
